# High-Performance All-Optical Terahertz Modulator Based on Graphene/TiO_2_/Si Trilayer Heterojunctions

**DOI:** 10.1186/s11671-019-2996-9

**Published:** 2019-05-10

**Authors:** Miaoqing Wei, Dainan Zhang, Yuanpeng Li, Lei Zhang, Lichuan Jin, Tianlong Wen, Feiming Bai, Huaiwu Zhang

**Affiliations:** State Key Laboratory of Electronic Thin Films and Integrated Devices, University of Electronic Science and Technology of China, Chengdu, 610054 People’s Republic of China

**Keywords:** THz modulator, TiO_2_ interlayer, Single-layer graphene, Built-in electric field, Broadband modulation

## Abstract

In this paper, we demonstrate a trilayer hybrid terahertz (THz) modulator made by combining a p-type silicon (p-Si) substrate, TiO_2_ interlayer, and single-layer graphene. The interface between Si and TiO_2_ introduced a built-in electric field, which drove the photoelectrons from Si to TiO_2_, and then the electrons injected into the graphene layer, causing the Fermi level of graphene to shift into a higher conduction band. The conductivity of graphene would increase, resulting in the decrease of transmitted terahertz wave. And the terahertz transmission modulation was realized. We observed a broadband modulation of the terahertz transmission in the frequency range from 0.3 to 1.7 THz and a large modulation depth of 88% with proper optical excitation. The results show that the graphene/TiO_2_/p-Si hybrid nanostructures exhibit great potential for terahertz broadband applications, such as terahertz imaging and communication.

## Introduction

Terahertz (THz) imaging technology [1] and terahertz communication technology [2, 3] are two major research directions in the field of THz. And the THz modulators are the basic components of the technologies, which can modulate the transmission and reflectivity of THz waves by modulating signals (light, electricity, heat, etc.) [4]. Much research has been done on THz modulators [5, 6], mainly focusing on materials. Semiconductor materials, such as Si and Ge, have been used for THz modulators. But the modulation performance is not ideal, and the modulation depth is not high, so many new materials have been proposed [7–9]. A representative new material is metamaterial. High-speed THz modulators can be realized by combining metamaterial with semiconductors. However, the bandwidth of the modulators based upon metamaterial is still very narrow due to the fixed structure and the fabrication process is complicated [10, 11]. Another typical material is a phase change material, such as VO_2_. At a certain temperature or voltage, the VO_2_ can undergo a reversible phase change between the insulating and metal states, and the electromagnetic properties change accordingly. The metallic state can cause an attenuation of the THz wave. But the THz wave can easily penetrate the insulating state of VO_2_. Therefore, the THz transmission can be modulated by applying external excitation to make the phase change of VO_2_. But such modulators [12–15] are based on the change of temperature, and have a slower temperature drop, so the modulation speed is slow.

In recent years, graphene has been gradually applied to THz technology due to its excellent electronic, optical, and mechanical properties [16–19]. Lee et al. fabricated an electrically controlled THz modulator by integrating graphene with metamaterials [20]. When electrical and optical properties of graphene were enhanced by the strong resonance of metal atoms, the light-matter interaction is enhanced, realizing the amplitude modulation of transmission terahertz wave by 47% and phase modulation by 32.2%. In 2012, Sensale et al. prepared a graphene-based field effect transistor (GFET) THz wave modulator, while the gate voltage tuned the carrier concentration in graphene [21]. However, the modulation depth of this kind of modulator [22–24] was shallow because of the limited carrier injection. The graphene/n-Si THz modulator prepared by Weis et al. has a modulation depth of up to 99% under the excitation of 808 nm femtosecond pulse laser [25]. Later, the graphene/n-Si THz modulator made by Li et al. achieved a modulation depth of 83% with simultaneous electrical and optical excitations. However, when no electric field was applied, only the light was added, and the modulation effect was not very well [26]. As a low-cost, non-toxic, and chemically stable semiconductor material, titanium dioxide (TiO_2_) has attracted great attention in the field of energy and environment. It is not only used for photocatalytic degradation of environmental pollutants, but also widely used in solar cells. Recently, Tao et al. prepared MoS_2_ film on TiO_2_ surface [27]. The interface introduced a strong built-in electric field, which enhanced the separation of electron-hole pairs, leading to the improvement of its photocatalytic properties. In 2017, Cao et al. made a high-performance perovskite/TiO_2_/Si photodetectors [28]. They attributed the improvement in performance to increased separation and reduced recombination of photoexcited carriers at the interface between Si and perovskite by the insertion of TiO_2_ film. Here, a graphene/TiO_2_/p-Si nanostructured all-optical THz modulator was fabricated. The device we designed has a large modulation depth of maximum 88% in the frequency range from 0.3 to 1.7 THz.

## Methods

The 500-μm-thick Si (p-type, resistivity *ρ* ~ 1–10 Ω cm) substrates were sequentially washed with acetone, ethanol, and deionized water for 20 min in an ultrasonic bath, and then immersed into 4.6 M HF solution for 10 min to remove the native oxide layer on the surface. Next, the cleaned Si was immersed into 0.1 M TiCl_4_ aqueous solution at 343 K for 1 h to obtain 10-nm-thick TiO_2_ film. Monolayer graphene was grown on copper by chemical vapor deposition [29]. And then, the graphene was transferred onto TiO_2_ film by using a wet etching method [30] to form graphene/TiO_2_/p-Si heterostructure. The entire sample area is 1 cm^2^. The quality of graphene was characterized by Raman spectroscopy. The absorption spectra were measured by a UV-visible spectrophotometer (Shimadzu, UV-3600). The ultraviolet photoemission spectroscopy (UPS) (Thermo Scientific, Escalab 250Xi) measurements were performed to get the energy band structure. The static modulation was evaluated by Fico THz time-domain system (Zomega Terahertz Corporation).

## Results and Discussion

**Fig. 1:**
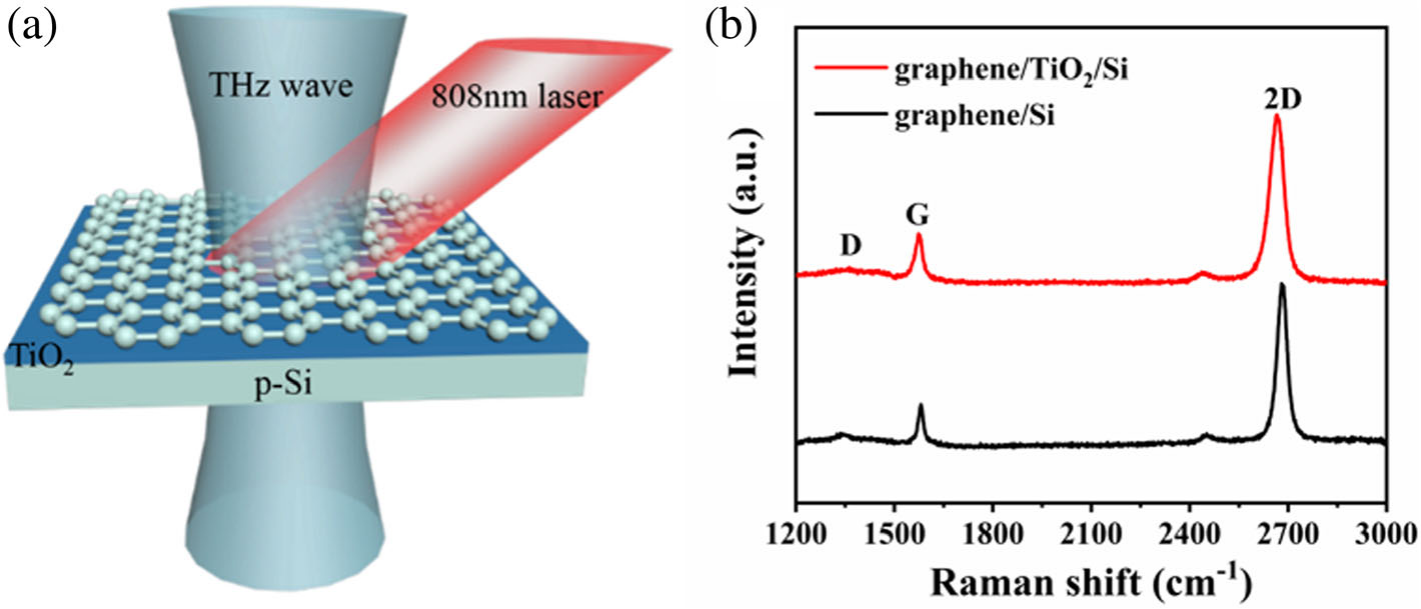
Experimental design and Raman spectra of graphene. **a** Schematic of the all-optical THz modulator. The modulator is composed of a single-layer graphene on a p-Si substrate with TiO_2_ film. **b** Raman spectra of the graphene on the Si and TiO_2_/p-Si substrates

The structure of an all-optical graphene/TiO_2_/p-Si THz modulator is depicted schematically in Fig. 1a. The THz wave and the laser were simultaneously incident from the graphene side. The semiconductor laser at a wavelength of 808 nm, spot diameter of ~ 5 mm, and power from 0 to 1400 mW was applied as the modulating signal. The THz beam (~ 3 mm) could be overlapped by the laser beam. And the transmitted THz waves were measured by a THz-TDS system at different laser powers. Because the performance of graphene modulators is relevant to the quality of graphene, we evaluated the quality of the transferred graphene on Si and TiO_2_/p-Si substrates by Raman spectroscopy with a 514-nm wavelength laser, as shown in Fig. 1b. It is obvious that the G peak and 2D peak of the graphene on p-Si are at ~ 1580 cm^−1^ and 2681 cm^−1^, respectively. For the graphene on TiO_2_/p-Si, the G peak is positioned at ~ 1575 cm^−1^ and the 2D peak is positioned at ~ 2667 cm^−1^. Compared with the Raman spectrum of graphene on silicon, the G and 2D peaks of graphene on TiO_2_/p-Si shift to the left because of the stress on graphene caused by the insertion of TiO_2_. In addition, the D peaks are weak for both the graphene on Si and TiO_2_/p-Si. The 2D peaks fit to a single Lorentzian and are more than twice the height of the G peaks for both of them. The Raman results indicate that the transferred graphene on Si and TiO_2_/p-Si is monolayer graphene with high quality [31].

**Fig. 2:**
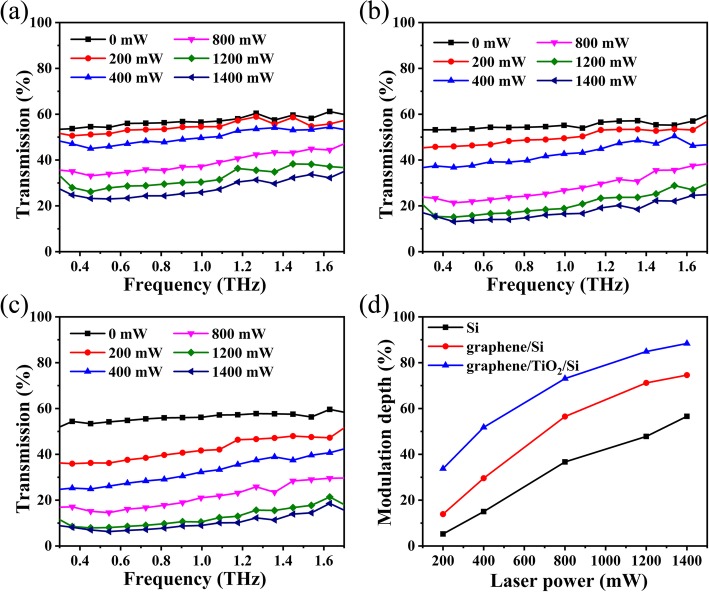
The modulation test. The transmittance spectra of the **a** Si, **b** graphene/p-Si, and **c** graphene/TiO_2_/p-Si at different laser power. **d** The modulation depth as functions of laser power for Si, graphene/Si, and graphene/TiO_2_/p-Si modulators

Figure 2a–c shows the THz wave transmittance of the Si, graphene/Si, and graphene/TiO_2_/Si at different laser power, respectively, which is measured by Fico THz time-domain system. Without photoexcitation, Si, graphene/Si, and graphene/TiO_2_/p-Si show a moderate transmission of ~ 55% of the THz wave due to the partial absorption and reflection from the carriers since the Si is p-doped. And the transmittances without photoexcitation have no notable difference for all of them, indicating the TiO_2_ and graphene do not attenuate the THz wave when there is no photoexcitation. Therefore, no additional insertion loss is caused by the TiO_2_ and graphene. When the power of the 808-nm laser increases from 0 to 1400 mW, the transmission decreases in the range of 0.3 THz to 1.7 THz for Si, graphene/p-Si, and graphene/TiO_2_/p-Si. When irradiated by laser with energy greater than the band gap of Si, electrons will be excited from the valence band to the conduction band. The excited electron-hole pairs will be formed on the surface, resulting in the increase of the conductivity. And the THz absorbance and reflectivity of semiconductors are dependent on the change of conductivity. Therefore, when THz wave penetrates through the Si irradiated by the laser, the intensity of the transmitted THz wave will decrease. What is more, the number of electron-hole pairs produced by Si under an 808-nm laser irradiation would increase as the laser power increases. And the increase of the conductivity of Si would result in the attenuation of the transmitted THz wave. In Fig. 2b, the transmission of graphene/Si decreases significantly with the increase of laser power than that of silicon. When the laser is irradiated onto the graphene/Si, the optical absorption in the Si is much higher than that in the graphene, so the number of generated carriers in the Si is much larger than that in the graphene. The free carriers will diffuse from silicon to graphene under the action of concentration gradient. Graphene has a higher carrier mobility and therefore undergoes a larger change in conductivity than Si. While the absorbance and reflectivity of THz depend on the change of conductivity, the modulation performance of graphene/p-Si is enhanced compared to Si. As shown in Fig. 2c, the transmission decrease of graphene/TiO_2_/p-Si is abrupt at the laser power of 200 mW and 400 mW. When the laser power continues to increase, the transmission decrease becomes milder. While the laser power applied is 1400 mW, the THz transmittance drops to around 10% in the range of 0.3 THz to 1.7 THz. The modulation depths can be calculated by (*T*_no excitation_ − T_excitation_)/*T*_no excitation_, where *T*_no excitation_ and *T*_excitation_ represent the intensity of THz transmission without and with photoexcitation, respectively. In order to more intuitively reveal its static modulation performance, we plotted the modulation depths as functions of laser power for Si, graphene/Si, and graphene/TiO_2_/p-Si, as shown in Fig. 2d. The modulation depth of graphene/Si is higher than that of Si, while the modulation depth of graphene/TiO_2_/p-Si is higher than graphene/p-Si. The modulation depths of all of them increase with increasing the laser power. When irradiated by 200 mW, the modulation depth of graphene/TiO_2_/p-Si is ~ 33%, about 6 times higher than Si, 2.5 times than graphene/Si, and higher than the THz modulators based on graphene field-effect transistors [21]. The modulation depth of graphene/TiO_2_/p-Si can reach 88% upon pumping by an 808-nm laser with a power of 1400 mW, higher than the graphene-based modulator with simultaneous electrical and optical excitations [26]. Therefore, from the static test, we can get the conclusion that the modulator is high performing with broadband and large modulation depth.

**Fig. 3:**
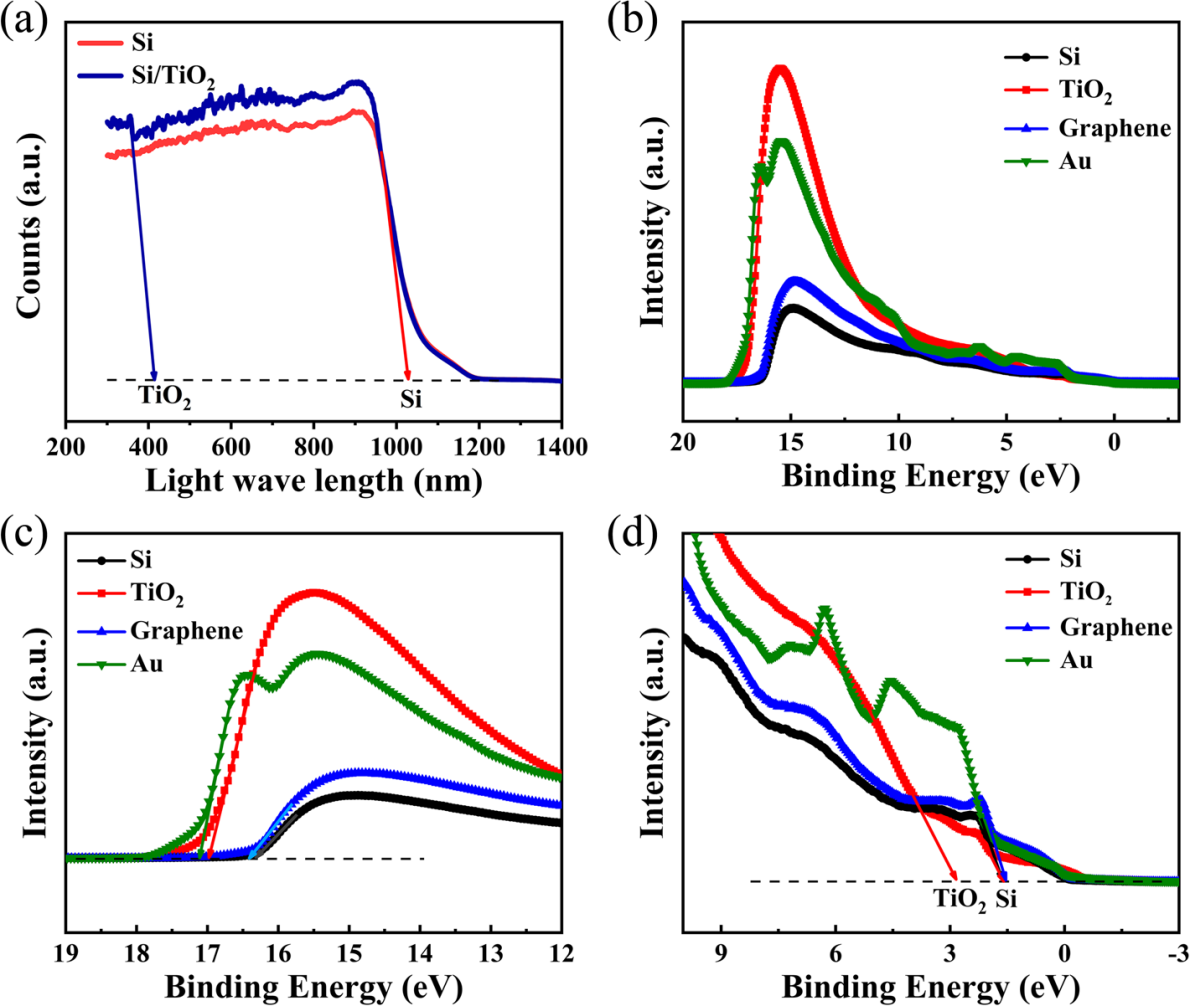
Absorption spectra and UPS spectra. **a** The absorption spectra of Si and TiO_2_/Si. **b** UPS spectra of Si, TiO_2_, graphene, and Au. **c** Enlarged parts of **b** showing the secondary electron onset. **d** Enlarged parts of **b** showing the valence band maximum

In order to obtain the energy band diagram of graphene/TiO_2_/Si modulator, we made the UV-visible spectrophotometer and the UPS measurements, as shown in Fig. 3. According to Fig. 3a, we can calculate that the band gap of Si and TiO_2_ is 1.19 and 2.98 eV, respectively. Figure 3b shows the UPS measurements on Si, TiO_2_, graphene, and Au. In order to confirm the meter’s Fermi level position, we performed the UPS measurements on Au [32]. And Fig. 3 c and d are the enlarged parts of Fig. 3b. From Fig. 3c, the secondary electron onset of the spectra is 16.33, 16.97, 16.43, and 17.11 eV for Si, TiO_2_, graphene, and Au, respectively. Therefore, the meter’s Fermi level position is 0.98 eV and the work function of Si, TiO_2,_ and graphene is calculated to be 5.85, 5.21, and 5.75 eV, respectively. According to Fig. 3(d), the value of valence band maximum of Si and TiO_2_ is located at 1.48 and 2.86 eV. The valence band level of Si and TiO_2_ is calculated to be − 6.35 and − 7.09 eV. Combining with the band gap of Si and TiO_2_, we can get the conduction band level of Si and TiO_2_, which is − 5.16 and − 4.11 eV.

**Fig. 4:**
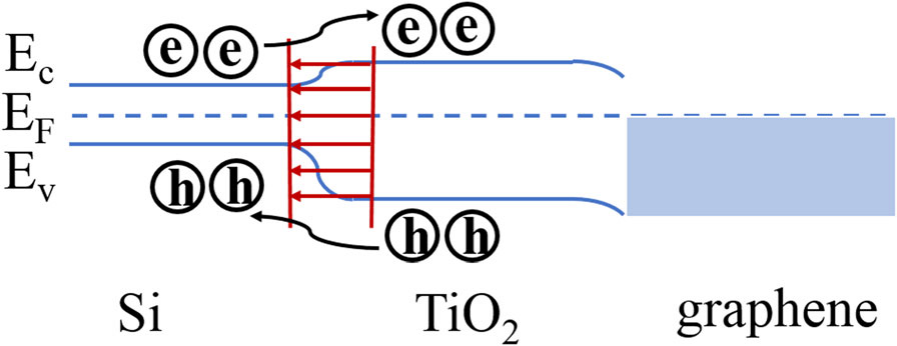
Band scheme of the graphene/TiO_2_/Si heterojunction

Based on the above results, the energy band diagram of the graphene/TiO_2_/Si heterojunction is illustrated in Fig. 4. E_c_, E_v_, and E_F_ denote the conduction band energy, the valence band energy, and the Fermi level energy, respectively. TiO_2_ is in direct contact with p-Si, and the electrons in TiO_2_ recombine with holes in p-Si, resulting in depletion layer at the interface. Since the TiO_2_ is “weaker” n-type, the depletion width in TiO_2_ is larger than in Si. Considering the TiO_2_ film is very thin (~ 10 nm), a fully depleted state would appear in the TiO_2_ layer. When graphene was transferred on TiO_2_/Si, there were no excess electrons in the TiO_2_ to migrate into the graphene. Therefore, there would be no carrier accumulation layer in dark state, and THz presented high transmission, which is consistent with the results in Fig. 2b. When the graphene/TiO_2_/p-Si heterojunction was photoexcited by the 808-nm laser, the amount of generated electron-hole pairs in Si was much larger than in graphene and TiO_2_. Upon photoexcitation, the Fermi level of the Si rose at the TiO_2_/p-Si interface. What is more, the electrons moved toward TiO_2_ and the holes toward Si due to the effect of the built-in electric field. The existence of TiO_2_ enhanced the separation of photoexcited carriers in Si, forming an n-type conductive layer in the thin TiO_2_ layer, hindering the transmission of THz wave. As the TiO_2_ layer is relatively thin, the effect on THz transmission is slightly less. After transferring graphene on TiO_2_/p-Si, a large number of electrons in TiO_2_ would be injected into graphene, which shifted the Fermi level into higher conduction band. Meanwhile, the conductivity of graphene increased, leading to higher attenuation of the THz wave. Thus, high modulation depth was realized.

## Conclusions

In summary, we have successfully fabricated a high-performance all-optical graphene/TiO_2_/p-Si terahertz modulator. The modulator exhibits broadband ranging from 0.3 to 1.7 THz, with 88% modulation depth. The inserting of TiO_2_ film introduced a PN junction with p-Si, and the built-in electric field enhanced the separation of photoexcited carriers in Si. The photoelectrons migrated from Si to TiO_2_, and then injected into the graphene layer, causing the Fermi level of graphene to shift into a higher conduction band. Therefore, the THz transmission modulation could be realized because of the increase of conductivity in graphene. The device is also very easy to make and low-cost. There is no need to deposit electrodes, and the TiO_2_ film can be prepared by a chemical solution method. What is more, the laser we used is a semiconductor laser, not necessarily the expensive femtosecond pulse laser as a modulation signal.

## Abbreviations

p-Si: P-type silicon
THz: Terahertz
UPS: Ultraviolet photoemission spectroscopy
